# Natural Variation in Sexual Traits and Gene Expression between Selfing and Outcrossing *Arabidopsis lyrata* Suggests Sexual Selection at Work

**DOI:** 10.1093/pcp/pcae090

**Published:** 2024-08-10

**Authors:** Ömer İltaş, Martin Čertner, Clément Lafon Placette

**Affiliations:** Department of Botany, Faculty of Science, Charles University, Benátská 2, CZ-128 01 Prague, Czech Republic; Department of Botany, Faculty of Science, Charles University, Benátská 2, CZ-128 01 Prague, Czech Republic; Department of Evolutionary Plant Biology, Institute of Botany of the Czech Academy of Sciences, Zámek 1, CZ-128 01 Průhonice, Czech Republic; Department of Botany, Faculty of Science, Charles University, Benátská 2, CZ-128 01 Prague, Czech Republic

**Keywords:** Natural variation, Pollen development, Pollen–pistil interactions, Selfing transition, Sexual gene expression, Sexual selection

## Abstract

Flowering plants show significant diversity in sexual strategies, profoundly impacting the evolution of sexual traits and associated genes. Sexual selection is one of the primary evolutionary forces driving sexual trait variation, particularly evident during pollen–pistil interactions, where pollen grains compete for fertilization and females select mating partners. Multiple mating may intensify competition among pollen donors for siring, while in contrast, self-fertilization reduces sire–sire competition, relaxing the sexual selection pressure. Traits involved in male–male competition and female choice are well described, and molecular mechanisms underlying pollen development and pollen–pistil interactions have been extensively studied in the model species *Arabidopsis thaliana*. However, whether these molecular mechanisms are involved in sexual selection in nature remains unclear. To address this gap, we measured intrinsic pollen performance and its interaction with female choice and investigated the associated gene expression patterns in a selfing and an outcrossing population of *Arabidopsis lyrata*. We found that pollen germination and pollen tube growth were significantly higher in outcrossers than selfers, and this difference was accompanied by changes in the expression of genes involved in vesicle transport and cytoskeleton. Outcrosser mother plants showed a negative impact on pollen tube growth compared to selfer mother plants, together with a difference of expression for genes involved in auxin and stress response, suggesting a potential mechanism for female choice through molecular cross talk at the post-pollination stage. Our study provides insight into the impact of sexual selection on the evolution of sexual gene expression in plants.

## Introduction

The evolution of sexual traits driven by sexual selection has been a long interest among biologists since Darwin’s (1871) first proposal. Sexual selection theory explains the evolution and persistence of favorable sexual traits associated with non-random success in competition for access to gametes (Alonzo and Servedio 2019, Shuker et al. 2021). It includes two selective forces: male–male competition, or intrasexual selection, where individuals of the same sex vie for the opportunity to mate, and female choice, or intersexual selection, where females select mates based on specific male traits. In plants, after the contact between pollen and pistil tissue (similar to mating in animals), sexual selection may occur through both male–male competition and female choice (Tonnabel et al. 2021). While sexual selection is now well accepted in plants (Moore and Pannell 2011), the genes underlying this evolutionary process remain understudied.

Upon landing on the stigma, pollen germinates and extends the sperm-carrying tube through the stylar tissue to fertilize the ovules (Johnson et al. 2019, Pereira et al. 2021). As the number of pollen grains received often exceeds the number of available ovules, this leads to competition among pollen donors for siring success (Christopher et al. 2020). Pollen germination and pollen tube growth are two significant traits likely to evolve under intra-sexual selection (Snow and Spira 1991, Delph et al. 1998, Mazer et al. 2010, Hove and Mazer 2013, Tonnabel et al. 2022). This assumption is supported by evidence suggesting that pollen donors with higher siring success demonstrate elevated germination rates (Austerlitz et al. 2012) and faster pollen tube growth (Snow and Spira 1996, Pasonen et al. 1999, Skogsmyr and Lankinen 2002) compared to those with lower siring success. The initial growth of the pollen tube is autotrophic and relies on precise gene regulation mechanisms, including vesicle transport, cytoskeleton dynamics, cell wall modification and Ca^2+^ signaling, to maintain continuous growth (reviewed in Cascallares et al. 2020, Scholz et al. 2020, Weng and Wang 2022). However, whether these molecular mechanisms are involved in sexual selection in nature remains unclear.

In addition to its autotrophic growth, a growing pollen tube communicates with the stylar tissue during its journey toward the ovules, a process known as pre-ovular guidance (Marshall et al. 1996, Mollet et al. 2000, Chae et al. 2007, Dresselhaus and Franklin-Tong 2013). The presence of numerous genetically distinct pollen donors on the stigma potentially allows the female gametophyte to exert selective force on tube growth abilities (Pannell and Labouche 2013). The female choice of superior sperm traits is well documented in animals at post-mating stage (namely cryptic female choice; Eberhard 1996) and represents similar aspects when considering the interaction between pollen and pistil tissue (Tonnabel et al. 2021). Female choice could influence mating outcomes directly by the molecular cross talk within the pistil or via the provisioning of elements required for pollen tube growth (Cheung et al. 1995, Park et al. 2000, Tonnabel et al. 2021). Nevertheless, while the molecular mechanisms involved in the interaction between the pollen tube and stylar tissues have been extensively studied, it remains to be investigated whether they contribute to female choice of conspecific mating partners in natural populations.

Several studies have found stronger footprints of positive and purifying selection on genes expressed in pollen than on sporophytic genes (Arunkumar et al. 2013, Gutiérrez-Valencia et al. 2022), which may be explained by sexual selection and/or haploid selection. Nevertheless, the developmental processes of pollen and pistil tissues are known to be highly conserved at the cellular and molecular levels (pollen: Wang et al. 2010; pistil: Zúñiga-Mayo et al. 2019). Therefore, genes involved in these processes are expected to exhibit pleiotropic effects (Rodríguez-Trelles et al. 2003, Signor and Nuzhdin 2018), potentially explaining the observed footprints of purifying selection on pollen genes (Arunkumar et al. 2013, Gutiérrez-Valencia et al. 2022). In contrast, positive selection is more likely to act on mutations with low pleiotropy, which may confine their detrimental effects (Connallon and Hall 2018). Pleiotropy might be alleviated by mutations occurring in cis-regulatory elements, which can restrict the mutational effect to cell type–specific gene expression. Indeed, most of the genetic variation underlying differences in gene expression is attributed to the effects on the activity of gene regulatory elements (Vuylsteke et al. 2005, Sicard et al. 2016). Therefore, sexual selection may favor the evolution of pollen performance and female choice traits through changes in gene regulatory elements. With transcriptome sequencing becoming more accessible, research on sexual selection is moving toward understanding the gene expression patterns evolving under selection (Wilkinson et al. 2015, Beaudry et al. 2020, Price et al. 2022, Tosto et al. 2023), a method well established in animals (Dean et al. 2015, Harrison et al. 2015, Veltsos et al. 2022), but yet to be tested in plants.

The diversity of reproductive strategies in plants provides valuable insights into how sexual selection operates in natural populations. For example, selfing species often mate with genetically identical partners, while outcrossing species primarily mate with genetically distinct partners. As a result, traits related to male–male competition and female choice may be relaxed in selfing lineages compared to their outcrossing counterparts (Skogsmyr and Lankinen 2002, Mazer et al. 2010, Wright et al. 2013, Cutter 2019). Consistently, several studies have shown that pollen tubes of outcrossing species tend to grow faster than those of selfing species (Smith-Huerta 1996, Taylor and Williams 2012, Hove and Mazer 2013, Mazer et al. 2018). Mating system shifts can also lead to significant genomic changes, potentially affecting how efficiently selection operates in selfing lineages. Due to higher levels of inbreeding, selfing species experience increased homozygosity, which reduces recombination efficiency and results in elevated linkage disequilibrium (Nordborg 1997, Wright et al. 2013). Although a shift in the mating system is known to play a profound role in the evolution of sexual traits, it may not be the sole driver. The evolution of sexual traits is likely to interact with local adaptation and is frequently influenced by multiple environmental factors acting as selective forces on genetic variation (Delph et al. 1997). For example, several studies have shown that environmental factors such as temperature and resource availability may also contribute to variation in pollen germination and pollen tube growth (Hedhly et al. 2004, Smith-Huerta et al. 2007, Liu et al. 2023a).

In this context, the transition to selfing may bear profound genomic consequences, including (but not restricted to) genes responsible for traits evolving under male–male competition and female choice. However, the transition to selfing leads to important long-term changes in sexual traits (‘selfing syndrome’) that are independent of sexual selection, bringing in a confounding factor when studying the impact of sexual selection on trait and gene evolution.

*Arabidopsis lyrata* appears as a relevant model system to test the effects of sexual selection on sexual traits and the evolution of gene expression without the confounding factor arising from the selfing syndrome. *Arabidopsis lyrata* is a perennial herbaceous plant with widespread distribution and made up of two recognized subspecies: *A. lyrata* ssp. *petraea* occurs in Eurasia, while *A. lyrata* ssp. *lyrata* occurs in North America (Al-Shehbaz and O’Kane 2002) (Ross-Ibarra et al. 2008, Schmickl et al. 2010). They vary in their habitat preferences, mating system and adaptation to their environment. European populations (*A. lyrata* ssp. *petraea*) are predominantly obligate outcrossers with a functional self-incompatibility system. They have a large population size and grow in open habitats including lakeshores, gypsum rock outcrops, scree slopes, cliffs and boulders (Jonsell et al. 1995, O’Kane and Al-Shehbaz 1997), as well as in semi-shaded places (Hemp 1996). It is assumed that the North American populations are the result of a colonization event from Europe that happened around 35,000 years ago (Ross-Ibarra et al. 2008). Colonization of the North American population was accompanied by a strong bottleneck (Ross-Ibarra et al. 2008, Schmickl et al. 2010), leading to genetically less diverse *A. lyrata* populations in North America (*A. lyrata* ssp. *lyrata*) compared with Europe (Schmickl et al. 2010). *Arabidopsis lyrata* ssp. *lyrata* occurs on sand dunes or rocky outcrops with some natural disturbance (Heblack et al. 2024). While most of the *A. lyrata* populations in North America (*A. lyrata* ssp. *lyrata*) are outcrossing, predominantly selfing populations with a loss of self-incompatibility have also been reported (Mable et al. 2005, Hoebe et al. 2009, Foxe et al. 2010). The breakdown of self-incompatibility occurred multiple times in populations located in the Great Lakes region and appears to be a recent event, occurring approximately 10,000 years ago (Foxe et al. 2010, Willi and Määttänen 2010). Due to this recent transition, North American selfing populations show no reduction in floral traits (aka ‘selfing syndrome’) compared to their ancestral outcrossing populations (Carleial et al. 2017). Moreover, the genome of *A. lyrata* has been already sequenced (Hu et al. 2011) and the annotation of the genome is well characterized using RNA-seq data (Rawat et al. 2015), making this species valuable to study consequences of the mating system shifts on the evolution of sexual traits and the underlying genes under sexual selection.

In this study, we used the two geographically isolated subspecies of *A. lyrata*, which differ in their mating systems, to test the following objectives: (i) whether pollen performance and pistil sorting ability upon pollination differ between them and (ii) whether the underlying genes responsible for pollen performance and female choice traits show variation in expression profiles between selfing *A. lyrata* ssp. *lyrata* and outcrossing *A. lyrata* ssp. *petraea* populations. For this purpose, we measured and compared pollen and female traits involved in intra- and intersexual selection between the selfing and outcrossing subspecies. In addition, we generated RNAseq data of pollen and pollinated-pistil tissues and identified differentially expressed genes (DEGs) between selfing and outcrossing *A. lyrata*. Our study shows variation in sexual traits and gene expression between selfing and outcrossing *A. lyrata* that is compatible with the effect of differences in the intensity of sexual selection.

## Results

### Concentration-dependent differences between outcrosser and selfer pollen performance

**Fig. 1:**
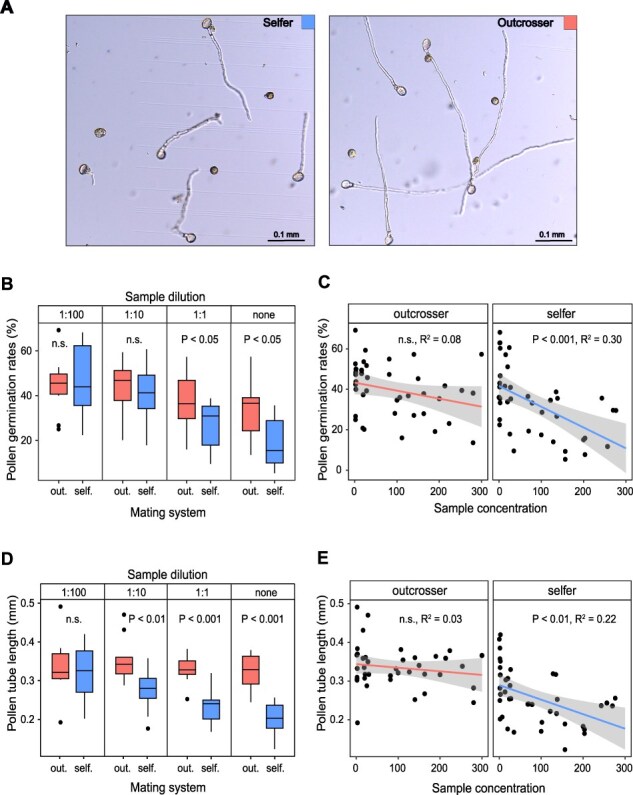
Higher performance of pollen originating from outcrosser than selfer individuals of *A. lyrata*. (A) Microscopy pictures showing the differences between the selfer and outcrosser pollen tube growth. (B, C) Pollen germination rates (%) and (D, E) pollen tube growth (mm) were assessed under experimentally manipulated gradients of pollen concentration resulting from different levels of sample dilution (1:100, 1:10, 1:1, none) after 4 h. Aside from plotting both response variables against the predictor pollen concentration (used in statistical models; **Table 1**), in (B) and (D) we provide alternative visualizations of the same relationships using sample dilution categories. All scale bars are 0.1 mm.

To test the phenotypic prevalence of gametophytic selection on pollen performance traits between the selfing *A. lyrata* ssp. *lyrata* and outcrossing *A. lyrata* ssp. *petraea*, we performed an in vitro liquid pollen germination assay and measured the pollen germination and pollen tube growth at an early (4 h) and late (16 h) time point (**Fig. 1A**). To experimentally simulate different levels of pollen competition for resources, we used a range of pollen concentration in the medium. At both time points, pollen concentration had a significant negative impact on germination (**Fig. 1B**, **Supplementary Fig. S4**, **Table 1**) and tube growth (**Fig. 1D**, **Supplementary Fig. S4**, **Table 1**). As a measure of pollen concentration, we tried using dilution factors or estimated pollen concentration values, and both approaches provided highly comparable results (**Fig. 1**).

**Table 1:** Summary of linear models testing the effects of the parental mating system (selfer vs. outcrosser), experimentally manipulated pollen concentration (a proxy of resource limitation) and their interactions on pollen performance

	d.f.	*F*	*P*	d.f.	*F*	*P*
A. Pollen germination rate						
LME model	Time = 4 h			Time = 16 h		
Pollen concentration	1, 66.4	34.5	**<0.001**	1, 48.2	14.9	**<0.001**
Mating system	1, 15.2	0.5	0.492	1, 6.4	1.5	0.258
Pollen concentration × Mating system	1, 66.4	7	**0.01**	1, 48.2	0.8	0.372
B. Pollen tube growth in vitro						
LME model	Time = 4 h			Time = 16 h		
Pollen concentration	1, 65.8	18.6	**<0.001**	1, 46.4	12.9	**<0.001**
Mating system	1, 14.7	5.5	**0.034**	1, 7.6	0.03	0.869
Pollen concentration × Mating system	1, 65.8	8.1	**0.006**	1, 46.4	1.9	0.172
C. Pollen tube growth in vivo						
ANOVA	Time = 10 h			Time = 16 h		
Maternal mating system (MMS)	1, 38	7.7	**0.009**	1, 60	6.5	**0.013**
Paternal mating system (PMS)	1, 38	6.9	**0.012**	1, 60	0.4	0.54
MMS × PMS	1, 38	0.5	0.488	1, 60	3.9	0.054

Pollen germination rates and pollen tube growth were established both in vitro under controlled conditions (A, B) and in vivo as pollen tube growth in pistils subjected to manipulated pollinations (C). Variation among different seed families was used as a random factor in LME models (A, B). Significant predictors and interactions (α = 0.05) are highlighted in bold.

At 4 h, both pollen germination and pollen tube growth were reduced with increasing pollen concentration in selfers, while the pollen performance in outcrossers was largely independent of pollen concentration, as reflected by the significant Pollen concentration × Mating system interaction in a linear mixed-effects (LME) model (**Fig. 1C**, **E**, **Table 1**). Consequently, the difference in pollen germination between outcrossers and selfers was most pronounced under high pollen concentrations and was least apparent under low pollen concentrations (**Fig. 1**). In addition, we observed a significantly faster pollen tube growth in outcrossers compared to selfers across the gradient of pollen concentration, as indicated by the significant effect of mating system in the LME model (**Fig. 1D**, **Table 1**).

At 16 h, pollen germination and tube growth were only affected by the pollen concentration, and no significant differences were detected between the selfer and the outcrosser (**Supplementary Fig. S4**, **Table 1**).

### Outcrossing mother plants show strong interference on pollen tube growth

**Fig. 2:**
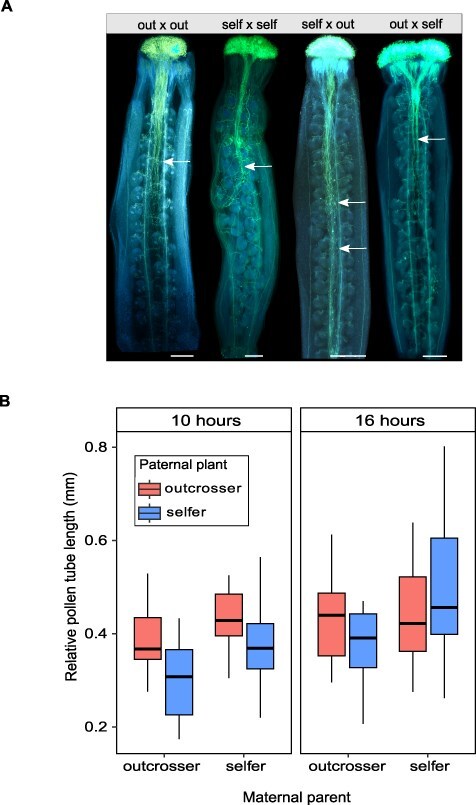
Male performance in pistils in experimental crosses testing the effects of the mating system (selfer vs. outcrosser) of both the maternal and paternal parent. (A) Aniline blue-stained pollinated pistil among the control and reciprocal crosses from the selfer and the outcrosser *A. lyrata* 16 h after pollination (cross directions always noted as ♀ × ♂). Arrows indicate the final point of the length of the majority of pollen tubes, forming a bulk. (B) Relative tube length among the crosses performed between paternal and maternal plants. Results are shown separately for two experiments differing in time allowed for pollen germination and growth (10 vs. 16 h). All scale bars are 0.25 mm.

We then tested the impact of the female tissues on pollen tube growth, under the hypothesis that female selfers and outcrossers would show different effects. For this purpose, we performed reciprocal crosses and measured pollen tube length at an early (10 h) and late (16 h) time point (**Fig. 2A**). Independently of treatment combination and experimental design, pollen tube length was significantly positively associated with the length of the pistil (**Supplementary Fig. S5**). Selfers and outcrossers did not differ in the pistil length (**Supplementary Fig. S5**). To account for variation in the pistil length among individuals and its effect on pollen tube length, we used relative pollen tube length (a ratio between absolute pollen tube length and pistil length) in all downstream analyses.

At 10 h, pollen of outcrossers developed longer tubes than pollen of selfers, independently of the mating system of the maternal plant (i.e. significant effect of paternal mating system in an analysis of variance (ANOVA) model; **Fig. 2B**, **Table 1C**). Additionally, outcrosser maternal plants had a negative impact on pollen tube growth compared to selfer maternal plants, independently of the pollen donor (significant effect of the maternal mating system in ANOVA; **Fig. 2B**, **Table 1C**). At 16 h, only the mating system of the maternal parent had an effect on pollen tube length, and pollen donor contribution was no longer apparent (**Fig. 2B**, **Table 1C**). We were only able to account for the effects of seed family variation in both maternal and paternal parents by fitting an LME model to the larger, combined dataset (10 + 16 h). This additional analysis confirmed the significant role of the mating system, even after including seed family information as random effects in the statistical model. Specifically, we found a significant Maternal mating system × Paternal mating system interaction, with the outcrosser maternal × selfer paternal crosses showing significantly lower pollen tube length than all other parental combinations (**Supplementary Table S5**).

### Pollen DEGs between the selfer and the outcrosser involve cytoskeleton and vesicle transport

**Fig. 3:**
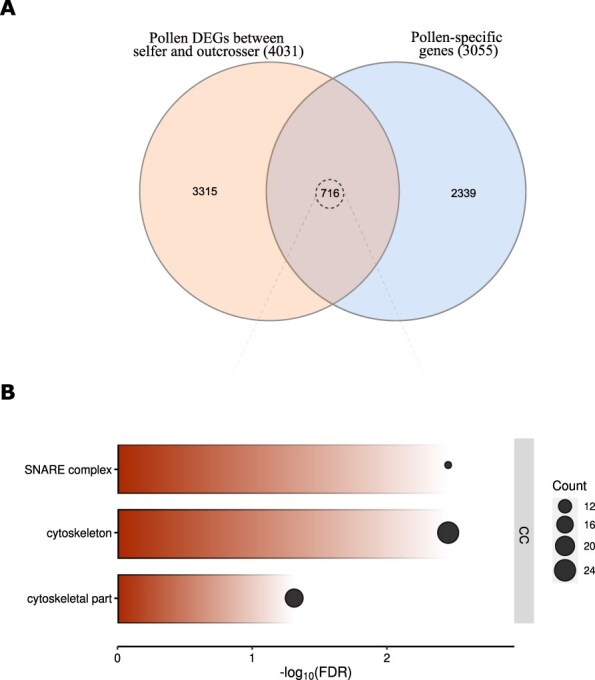
Candidate pollen-specific genes together showing divergent expression between selfing and outcrossing plants with their biological functions. (A) Venn diagram showing the number of candidate genes under the sexual selection in pollen (716 DEGs) and (B) GO term enrichment analysis of those candidate genes in pollen within the biological process (FDR <0.05 and *q* < 0.05). The size of the black bubble refers to the number of genes enriched in each category.

We searched for gene expression patterns associated with the difference in pollen traits observed between selfers and outcrossers (**Fig. 1**). To do so, we first identified genes preferentially expressed in pollen by performing a differential expression analysis (DEA) between pollen germinated in vitro and other tissues (leaves, roots, emasculated pistils and endosperm; see methods for the criteria we used; **Supplementary Fig. S2A**). A total of 3,055 DEGs were detected from this comparison and used as pollen-specific genes for further analysis. To validate biological functions of pollen-specific genes, we performed Gene Ontology (GO) enrichment analysis. ‘Pollen tube growth’, ‘pollination’, ‘pollen tube development’, ‘multi-organism reproductive process’ and ‘cell tip growth’ were some of the highly represented GO terms within the biological process (False Discovery Rate, FDR <0.05 and *q* < 0.05; **Supplementary Fig. S3**). Then, to find pollen genes differentially expressed between pollen from the outcrosser and the selfer subspecies, we performed a DEA between the outcrosser and selfer pollen. A total of 4,031 genes were identified as differentially expressed with 2,033 of them being downregulated and 1,998 being upregulated in outcrosser compared to selfer pollen. As many of these genes are likely to be expressed in other tissues than just pollen and thus to have a general function, we aimed at narrowing down the genes responsible for pollen performance differences between selfers and outcrossers. To do so, we overlapped the pollen-specific genes (3,055) with pollen DEGs between selfers and outcrossers (4,033 genes). By doing so, we identified 716 genes (**Fig. 3A**). These genes showed three significantly enriched GO terms (FDR <0.05 and *q* < 0.05; **Supplementary Table S6**), namely SNARE (soluble NSF attachment protein receptor) complex (a complex of proteins drives the fusion of vesicles with the target membrane), cytoskeleton and cytoskeletal parts (**Fig. 3B**).

### Pollinated pistil DEGs show enriched functions in auxin and stress signaling

We finally searched for genes associated with the differential maternal plant response to pollen between selfers and outcrossers (**Fig. 2**). To characterize the transcriptome profile of pollen and pistil interaction, we first identified genes that are preferentially expressed in pollinated pistils (within-mating system pollination only). We performed a DEA between pollinated pistil and the tissues composing it (pollen, emasculated pistils and endosperm as markers for already formed seeds) and detected 208 genes as pollinated pistil–specific genes (see methods for criteria; **Supplementary Fig. S2B**). Among these pollinated pistil–specific genes, several enriched GO terms were found, including ‘response to organic substance’, ‘response to chemical’, ‘defense response’ and ‘response to stimulus’ (FDR <0.05 and *q* < 0.05; **Supplementary Fig. S3**).

**Fig. 4:**
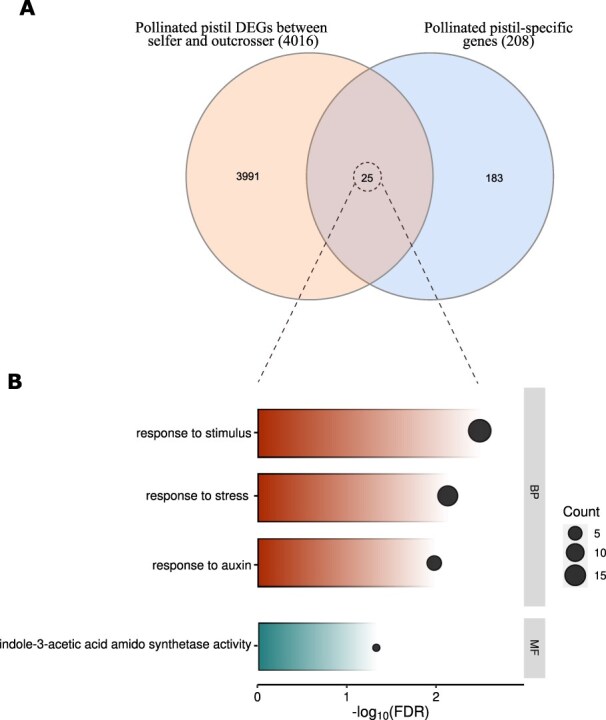
Candidate pollinated pistil–specific genes together showing divergent expression between selfing and outcrossing plants with their biological functions. (A) Venn diagram shows the number of pollinated pistil candidate genes (25 DEGs) for the sexual selection and (B) GO term enrichment analysis of those candidate genes in pollinated pistil within the biological process and molecular function (FDR <0.05 and *q* < 0.05). The size of the black bubble refers to the number of genes enriched in each category.

Furthermore, we performed a DEA between the selfer- and outcrosser-pollinated pistils to identify the candidate genes associated with mating system–dependent maternal response to pollen. In total, 4,016 genes were detected, among which 1,876 were upregulated and 2,140 downregulated (see methods for the criteria). As many of these genes are likely expressed in other tissues and are thus not related to pistil–pollen interactions, we narrowed down the list of candidates by overlapping pollinated pistil–specific genes with DEGs obtained between the selfer- and outcrosser-pollinated pistils. A total of 25 genes were found (**Fig. 4A**). These genes showed significantly enriched GO terms such as ‘response to stimulus’, ‘response to stress’, ‘response to auxin’ and ‘indole-3-acetic acid amido synthetase activity’ (**Fig. 4B**). The gene list is available in **Table 2**.

**Table 2:** Detailed list of 25 pollinated pistil–specific candidate genes showing divergent expression between selfing and outcrossing plants and their description and expression values

*Arabidopsis lyrata* gene name	*Arabidopsis thaliana* ortholog	Description	log2FoldChange	*P*_adj_	Regulation
AL1G66590	AT1G56650	Production of anthocyanin pigment 1	2.27738456	4.01E-05	Up
AL1G34180	AT1G21130	*O*-methyltransferase family protein	5.771687311	4.09E-05	Up
AL4G27410	AT2G32030	Acyl-CoA *N*-acyltransferases superfamily protein	3.124196917	4.57E-05	Up
AL6G21160	AT5G10625	NA	2.389417277	0.000114949	Up
AL4G10820	AT2G21620	Adenine nucleotide alpha hydrolases–like superfamily protein	1.117985273	0.000248226	Up
AL6G47870	AT4G04330	Chaperonin-like RbcX protein	−2.529241661	0.000334042	Down
AL1G33580	AT1G20620	Catalase 3	−1.626009186	0.000400621	Down
AL3G52410	AT1G69730	Wall-associated kinase family protein	5.682140958	0.00053256	Up
AL5G44800	AT3G62150	*P*-glycoprotein 21	3.741307124	0.000636484	Up
AL5G14590	AT3G25930	Adenine nucleotide alpha hydrolases–like superfamily protein	2.106417629	0.000715791	Up
AL8G24230	AT5G50200	Nitrate transmembrane transporters	2.362197731	0.000916552	Up
AL6G50520	AT4G02520	Glutathione *S*-transferase PHI 2	2.535846662	0.00103574	Up
AL1G22630	AT1G11530	C-terminal cysteine residue is changed to a serine 1	1.585825356	0.001559205	Up
AL1G41660	AT1G28130	Auxin-responsive GH3 family protein	1.872423364	0.002825036	Up
AL2G27690	AT1G68760	Nudix hydrolase 1	−0.983314842	0.003276667	Down
AL8G28640	AT5G53730	Late embryogenesis abundant hydroxyproline-rich glycoprotein family	1.496028194	0.006214946	Up
AL7G21490	AT4G30650	Low-temperature and salt-responsive protein family	1.973107626	0.008353171	Up
AL4G27150	AT2G31865	Poly (ADP-ribose) glycohydrolase 2	2.8765681	0.012083748	Up
AL1G54460	AT1G47960	Cell wall/vacuolar inhibitor of fructosidase 1	−2.235527378	0.013239139	Down
AL8G14450	AT5G44990	Glutathione *S*-transferase family protein	2.993517576	0.016405063	Up
AL1G16470	AT1G06460	Alpha-crystallin domain 32.1	1.046839627	0.017572315	Up
AL3G51340	AT2G18150	Peroxidase superfamily protein	2.349665778	0.027822045	Up
AL3G36350	AT3G22160	VQ motif-containing protein	2.201899453	0.037081683	Up
AL4G13030	AT2G23170	Auxin-responsive GH3 family protein	1.996218442	0.040542136	Up
AL4G27560	AT2G32200	NA	1.4652235	0.049394014	Up

Descriptions of genes retrieved from the *A. lyrata* v2.1 annotation file (Rawat et al. 2015).

## Discussion

### The differences in sexual traits between selfing and outcrossing *A. lyrata* are consistent with the effect of sexual selection

In this study, we tested whether male performance and female choice differ between selfing and outcrossing *A. lyrata*. We assumed that these two traits would show a reduction in the selfing lineage compared to the outcrossing one due to relaxed sexual selection (Mazer et al. 2018, Cutter 2019). By combining in vitro and in vivo (reciprocal crosses) pollen germination experiments, we could separate the pollen’s intrinsic abilities from the impact of female tissues on pollen tube growth.

Through in vitro pollen germination assays, we assessed the inherent abilities of pollen donors independently of female gametophyte interaction. Our findings revealed significantly higher pollen germination and pollen tube length in the outcrosser compared to their selfing counterparts, particularly at the early time point (4 h). These in vitro results mirror the in vivo findings from controlled crosses, showing that outcrosser pollen had longer tubes than the selfer one at the early time point (10 h). To date, despite theoretical predictions on the impact of mating system shifts on male competition ability (Mazer et al. 2010, Lankinen and Karlsson Green 2015, Cutter 2019), empirical studies have been limited, primarily focusing on Clarkia species (Németh and Smith‐Huerta 2003, Hove and Mazer 2013, Mazer et al. 2018). Especially, how fast a transition to selfing can lead to divergence in pollen performance traits between sister lineages remains largely unexplored. It is known that the divergence between the Eurasian and North American lineages occurred around 35,000 years ago (Ross-Ibarra et al. 2008). The breakdown of self-incompatibility in North American populations appears to be a more recent event, occurring approximately 10,000 years ago (Foxe et al. 2010), making *A. lyrata* a valuable model system for studying recent transitions to selfing. Our study shows that pollen performance can rapidly decline following the transition to selfing. This decline seems to have appeared faster than and independently from the ‘selfing syndrome’, which is not visible in the selfing *A. lyrata* lineage we used (Carleial et al. 2017). The difference in pollen performance between selfing and outcrossing *A. lyrata* was the most pronounced in higher pollen concentration in the liquid medium. Higher pollen concentration is likely to be associated with stronger competition for nutrients present in the medium and may to a certain extent mimic pollen–pollen competition for the nutrients provided by female tissues (Erbar 2003). In this context, outcrosser pollen may perform better in more competitive environments than selfer pollen, which may be explained by differences in the intensity of sexual selection between the two lineages (Cutter 2019).

By performing reciprocal crosses, we could identify a divergent role of female selfers and outcrossers on pollen tube growth. It is important to note that our experimental design did not allow us to test the impact of individual maternal plants on pollen tube growth at each time point separately, which could be important. Larger scale studies should be considered, even though conducting microscopy work at a larger scale would be tedious and time-consuming. Nevertheless, our findings suggest that female outcrossers have a significant and negative impact on pollen tube growth compared to female selfers, independently of the pollen donor. Variation in pollen tube length due to maternal genotype interference may stem from molecular interactions between the growing pollen tube and stylar tissue at post-pollination stage, potentially serving as a substrate for sexual selection (Tonnabel et al. 2021). As female tissues provide nutrients for pollen tubes to grow (Johnson and Preuss 2002, Chae and Lord 2011, Pereira et al. 2021), female outcrossers may have evolved to restrict their provisioning to select for the most vigorous pollen grains.

With this in mind, we are aware that our comparison involved only one outcrossing population and one selfing from two geographically isolated subspecies: *A. lyrata* ssp. *petraea* (outcrossing) occurs in Eurasia, while *A. lyrata* ssp. *lyrata* (selfing) occurs in North America (Al-Shehbaz and O’Kane 2002), and observed differences could be due to other factors such as lineage divergence, genetic drift or other selective processes (like ecological differences) rather than sexual selection. The interaction between ecological factors and sexual selection is noteworthy, as the environmentally plastic nature of pollen performance is likely to impact the efficacy of selection acting on it (Delph et al. 1997) and could explain our results, as the two populations we used show ecological differences. A further study should include North American outcrossers closely related to and geographically located near the selfers (Li et al. 2023) and/or a broader range of independent lineages to build stronger conclusions, such as the recently discovered selfing *A. lyrata* in Siberia (Kolesnikova et al. 2023). Nevertheless, the higher pollen performance and more strict female influence on pollen tube growth we observed in the outcrossers are consistent with the impact of sexual selection. Independently of this conclusion, our findings provide a system involving natural variation in pollen and female traits that may prove useful to understand the molecular mechanisms underlying the evolution of these traits in nature.

### Mating system shift differences in pollen performance mirror differential expression of genes important for pollen development

To uncover the molecular mechanisms behind the pollen performance differences we observed between selfers and outcrossers, we compared their pollen gene expression profiles. We identified 716 genes being both pollen-specific and differentially expressed between selfer and outcrosser *A. lyrata*. Among these genes, significantly enriched functions were associated with the SNARE complex, cytoskeleton and cytoskeletal parts, reflecting biological functions important for pollen development.

Indeed, these processes are vital for efficient pollen tube growth during fertilization. Pollen tube growth is characterized by polar tip growth and is regulated by cytoplasmic streaming during the synthesis of a new cell wall at the tip region (Lovy‐Wheeler et al. 2007). Active streaming of cell wall materials, enzymes and signal molecules to the growing tip region, facilitated by secretory vesicles, is essential for pollen tube growth (Ruan et al. 2021, Weng and Wang 2022). The SNARE complex regulates intracellular membrane fusion events, facilitating the delivery of necessary cell wall materials for expansion and elongation, promoting pollen tube growth toward the ovule for fertilization (Guo and McCubbin 2012, Slane et al. 2017, Macgregor et al. 2023, Liu et al. 2023b). Several SNARE proteins are specifically expressed in pollen tissue, with some located at the pollen tube apex and behind the tip, as well as on the plasma membrane of pollen tubes (Enami et al. 2009, Ichikawa et al. 2014, Slane et al. 2017). It has been shown that knock-out mutations in the SNARE complex cause severe defects in pollen tube growth within the style (Slane et al. 2017). Additionally, the essential role of the cytoskeleton and its components in organizing the trafficking of endo- and exocytotic vesicles during pollen tube growth is well understood and has been shown by numerous studies (Cole and Fowler 2006, Qin and Yang 2011, Xu and Huang 2020). As the genes involved in these processes were differentially expressed between selfing and outcrossing *A. lyrata*, this suggests that sexual selection may act on the expression of these genes. Previous population genomics studies estimated the molecular evolutionary rate of pollen-expressed genes in selfing (Harrison et al. 2019) and outcrossing (Arunkumar et al. 2013, Gutiérrez-Valencia et al. 2022) populations. While informative, these studies do not pinpoint specific traits and genes essential for intra-sexual selection. In our study, we used species-specific and pollen-specific transcriptomics and identified candidate genes involved in pollen tube growth in outcrosser *A. lyrata*, potentially evolving under strong intra-sexual selection pressure at the post-pollination stage. Finally, the enriched functions we found only included 56 genes attributed to these functions from *A. thaliana* annotation. As this annotation is largely based on the observation of mutants in lab conditions, the 661 remaining genes from our transcriptomic study may be unforeseen candidates responsible for natural variation in pollen traits in the wild, presenting opportunities for future research.

### Female choice may operate as a defense response mechanism following pollination

Upon germination on the stigma, the pollen tube receives external signals from the stylar tissue, guiding its growth toward the ovule (pre-ovular guidance; reviewed in Cheung et al. 1995, Park et al. 2000, Higashiyama and Yang 2017). This finding has drawn attention to possible mechanisms for pistil traits to discriminate among preferable pollen donors, similar to cryptic female choice in animals (Tonnabel et al. 2021). However, studies focusing on the molecular mechanisms of pollen and pistil interaction often utilize knock-out approaches to identify genes, overlooking selection pressures in natural populations. In this context, we aimed at finding differential gene expression profiles involved in pollen–pistil interactions upon pollination from natural outcrossing and selfing populations. We found 25 genes both being pollinated pistil–specific and differentially expressed between outcrossers and selfers, representing potential candidates for female choice. Among these, enriched functions were related to auxin synthesis and response as well as stress response.

Multiple studies highlight the importance of auxin and its derivatives [indole-3-acetic acid amido (IAA)] in pollen germination and tube growth following pollination. For instance, Aloni et al. (2006) observed elevated auxin levels in the pollen tube tip and beneath the stigma, suggesting their role in regulating pollen germination and tube growth. Similarly, Chen and Zhao (2008) emphasized the significance of free IAA in stylar tissue during pollen tube growth after pollination. Moreover, they discovered elevated IAA levels in the apical and middle parts of the style, correlating with enhanced pollen tube growth. In this context, pistil tissue with differential IAA expression and auxin response in the stylar region may function as a mechanism for female choice, spatially regulating IAA production to allow pollen donors to display their performance. Another recent study by Chebli and Geitmann (2024) demonstrated the invasive growth of pollen tubes in the pistil, revealing that the enzyme pectate lyase–like (PLL), secreted by pollen tubes, digests pectin from the transmitting tissue of the pistil to facilitate its growth. This may explain why pollen–style interactions after penetration resemble a pathogen response at the molecular level (Elleman and Dickinson 1999, Dresselhaus and Márton 2009, Shi et al. 2017). The transcriptome of pollinated pistils in our study also revealed enriched functions associated with defense response. Although direct evidence of pistil counteraction against the invasive growth of the pollen tube is lacking, it is known that pistil secretes proteins that interact with the pollen tube (Cheung et al. 1995, Park et al. 2000). These pistil-secreted molecules may reduce pollen invasiveness by affecting PLL activity (Sanati Nezhad and Geitmann 2013). As pistil genes differentially expressed between selfers and outcrossers included enriched function in stress response in our study, we hypothesize that female choice, and the more restrictive influence of female outcrossers on pollen tube growth, may act through a similar pathway as a pathogen response against growing pollen tubes. Further studies may test this hypothesis.

In conclusion, we found that outcrossers, experiencing strong intra-sexual selection in nature, showed higher pollen performance and stricter female choice than selfers. We revealed candidate genes underlying this trait divergence with a transcriptomic approach, paving the way for further studies on the genomics of sexual selection in plants, a largely unexplored field.

## Material and Methods

### Plant material and cultivation conditions

In this study, we used individuals from two subspecies of *A. lyrata* that are known to differ in their mating system, one selfing and the other outcrossing. Seeds from the selfer population (*A. lyrata* ssp. *lyrata*) were collected from Point Pelee, ON, Canada (GPS coordinates: 41°55ʹ40.0″N, 82°30ʹ58.0″W), and seeds from the outcrosser population (*A. lyrata* ssp. *petrea*) were collected from Nová Ves near Oslavany, Czech Republic (GPS coordinates: 49°07ʹ03.1″N, 16°18ʹ21.4″E). Prior to germination, seeds were surface-sterilized using a sterilization solution [consisting of 5% NaClO, 0.01% (v/v) Triton X-100, and sterile water] and planted on agar plates containing MS-Salts, MES hydrate and 0.8% (w/v) plant agar with pH 5.8. Plates were then transferred to a growth chamber with the following conditions: a 12-h day at 23°C and a 12-h night at 13°C, and cultivated for 2 weeks. After that, seedlings were transplanted into small pots filled with garden substrate and grown for 8 weeks under short-day conditions (8 h of light per day at 4°C) to induce flowering. Finally, young plants were repotted into 0.5-l pots, transferred to a growth chamber and cultivated under long-day conditions (a 16-h day at 21°C, a 8-h night at 15°C) until they were used in experiments.

### In vitro pollen germination and pollen tube growth assay

To test the intrinsic pollen performance abilities, we performed an in vitro liquid pollen germination assay using pollen from selfer and outcrosser subspecies of *A. lyrata*. The assay was performed following an original protocol adapted from Xiyan (2011) with some modifications outlined later. For all experiments, for feasibility reasons, we prioritized the number of mother plants over the number of replicates per mother plant. This way, we could catch a larger amount of genetic diversity to have a representative sampling of each population, with the drawback that the low number of replicates per mother plant did not allow us to test the effect of mother plants.

Initially, we performed a pilot experiment to determine the timeline of pollen tube growth, as well as to assess the impact of different conditions on pollen germination. Time was indeed the most relevant difference for this experiment and other distinctions were rather technical, such as volume in which germination took place and dilution. We began by using 2-ml tubes for pollen incubation and allowing the pollen to germinate for 16 h at room temperature (RT) to ensure sufficient time for germination. For this pilot experiment, we used four mother plants (with two to four offspring per mother as individual replicates) for both the selfer and the outcrosser.

Subsequently, we decided to refine the assay conditions by reducing the incubation time to 4 h at 25°C to examine the early response of intrinsic pollen performance abilities. Moreover, we transitioned to using 15-ml Falcon tubes to ensure ample oxygen availability for pollen tube respiration compared to the limited oxygen supply in the 2-ml tubes. For this experiment, we used four mother plants (with one to two offspring per mother as individual replicates) for both the selfer and the outcrosser.

To start the pollen germination assay, 15 freshly opened flowers from the apical inflorescence were collected in 2-ml tubes, and chilled at RT for 30 min, with the tube cap remaining open. Subsequently, 1 ml of freshly prepared germination medium (containing 5 mM MES-Tris adjusted to pH 5.8, 1 mM KCl, 0.8 mM MgSO_4_, 1.5 mM boric acid, 10 mM CaCl_2_, 5% w/v sucrose and 15% w/v PEG4000) was added to the tubes and vortexed at maximal speed for 1 min. Then, pollen grains were concentrated by centrifugation at 6,000 rpm for 5 min at RT. The supernatant and flower residues were removed, and the pollen pellets were resuspended in 1 ml of germination medium by vortexing. The concentration of pollen grains was measured using 10 μl of pollen suspension placed on a hemocytometer counting chamber under a light microscope. Subsequently, pollen suspensions were diluted in serial batches (including no dilution, 1/2, 1/10 and 1/100) in 15-ml falcon tubes for the 4-h experiment and 2-ml tubes for the 16-h pilot experiment to test pollen performance under resource limitation. Following that, pollen suspensions were incubated at 25°C for 4 h or at RT for 16 h with 250 rpm agitation. Finally, 10 μl of the germinated pollen suspension was placed on a hemocytometer counting chamber and photographed by using differential interference contrast microscopy with an Olympus BX51 (Olympus Corp., Tokyo, Japan) equipped with Canon EOS 700D camera (Canon Inc., Tokyo, Japan).

Pollen germination and tube length were analyzed using the ImageJ program (https://imagej.nih.gov/ij/) in a double-blind manner. Pollen grains were considered germinated when a pollen tube emerged and its length was equal to or greater than the pollen grain diameter. The germination rates (%) were calculated by dividing the number of germinated pollen grains by the total number of pollen grains per sample. The length of the pollen tube (μm) was measured using the segmented line option in the ImageJ program, and the mean pollen tube length was determined as the average length of 30 germinated pollen grains. Detailed measurements can be found in **Supplementary Table S1**.

### *In vivo* pollen tube growth assay

To assess the female action on pollen tube growth, we performed control and reciprocal crosses between selfers and outcrossers of *A. lyrata*. For this experiment, we again used two different time points to observe pollen tube growth at 10 and 16 h after the pollination. Each time point was done in a separate experiment using a different set of maternal and paternal individuals to perform manual crosses (see **Supplementary Table S2** for details). For each designated cross and time point, we performed 10–15 manual pollinations for each cross-type (♀ × ♂; selfer × selfer, outcrosser × outcrosser, outcrosser × selfer and selfer × outcrosser). Between two to four different maternal plants and two to four different paternal plants were randomly combined for each type of cross (**Supplementary Table S2**). For outcrosser crosses, we first did a test cross to see whether plants were compatible and could produce fruits to avoid crossing plants with the same self-incompatibility allele that would result in no pollen tube growth. For all crosses, flowers were emasculated before anthesis, and pistils were hand-pollinated 2 d after emasculation. Pollinated pistils were collected after 10 and 16 h of pollination and stored in a fixation solution (containing acetic acid/EtOH, 1:3 ratio) for 1 d at RT. After fixation, the fixed pistils were rehydrated through a series of ethanol dilutions using 70%, 50% and 30% ethanol series, respectively, and washed with ddH_2_O. Pistils were softened overnight at RT using 8M NaOH and subsequently stained with a decolorized aniline blue solution (0.1% w/v aniline blue in 108 mM K_3_PO_4_, pH 11) for 3 h in the dark. Stained pistils were observed and pictured under UV light conditions using a fluorescence Olympus BX51 microscope (Olympus Corp., Tokyo, Japan) equipped with a Nikon Digital Sight 10 camera (Nikon Instruments Inc., Melville, NY, USA). Finally, images were analyzed using the ImageJ program (https://imagej.nih.gov/ij/). The length of the majority of pollen tubes, forming a bulk, was measured as a mean value for pollen tube length. All measurements were conducted in a double-blind manner.

### Statistical analyses of pollen tube growth

LME models were used to compare the differences in pollen germination and pollen tube growth between selfer and outcrosser individuals. Separate models were fitted for different designs of experiments, differing in time at which the male performance was assessed and using different sets of *A. lyrata* individuals (recruited from mostly non-overlapping suites of seed families). The mating system of an individual (selfer and outcrosser), in case of manipulated crosses for the maternal and paternal parent separately, pollen concentration (in vitro experiments only) and their interactions were used as fixed effects in the models. Seed family (nested within the mating system) was used as a random effect in the models. LME models of the Gaussian family were fitted with the restricted maximum likelihood method as implemented in the R package ‘lme4’ ver. 1.1-34 (Bates et al. 2015). The response variable Pollen tube growth in vitro was log-transformed for the 16-h dataset to meet the model assumptions. Statistical significance was inferred in a type III analysis of variance with Satterthwaite’s method to estimate the denominator degrees of freedom using the R package ‘lmerTest’ (Kuznetsova et al. 2017).

When analyzing the dataset of in vivo pollen performance in manipulated crosses, incorporating the seed family identity for both maternal and paternal parents as random effects resulted in too complex LME models (singular fit) given the number of observations available within each of the two experimental designs (time = 10 h, 16 h). Therefore, we instead used two-way ANOVA models to analyze the two designs separately. In addition, we applied an LME model to the combined dataset of both designs, with maternal and paternal seed families as random effects, to check the consistency of the main results after including random effects in the statistical model.

Simple linear models (*t*-test, least-squares regression) were applied to illustrate differences between compared groups and the strength of relationships plotted in graphs. Unless stated otherwise, statistical data analysis was done with R ver. 4.3.1 (R Core Team 2023).

### RNA extraction, library preparation and sequencing

We isolated total RNA from sexual and non-sexual tissues from both outcrossers and selfers of *A. lyrata* (see **Supplementary Table S3** for details). For each tissue, we collected material from one individual per mother plant from four different mother plants for both the selfer and outcrosser subspecies (2 subspecies × 4 mother plants × 1 individual = 8 samples sequenced per tissue in total). In total, we sequenced 40 samples (2 subspecies × 5 tissues × 4 mother plants × 1 individual) for the transcriptomic analysis.

Non-sexual tissues included leaves and roots, and sexual tissues consisted of pollen, emasculated unpollinated pistils, and pollinated pistils. For emasculated pistils, we first emasculated the flowers before anthesis, then collected 10 pistils 2 d after emasculation and immediately froze them in liquid nitrogen until the day of RNA extraction. For pollinated pistils, we first emasculated the flowers before anthesis. Two days after emasculation, we then performed 10 manual control crosses (selfer × selfer and outcrosser × outcrosser) and collected pollinated pistils 6 h after pollination by immediately freezing them in liquid nitrogen until the day of RNA extraction. To extract RNA from germinated pollen, a total of 60–100 freshly opened flowers were collected from a single offspring of four different mother plants for both selfing and outcrossing populations (eight plants in total). A single plant cannot produce this amount of flowers simultaneously, and therefore we collected flowers >3 d to ensure having 60–100 flowers per plant. Pollen was extracted from the flowers and germinated in a liquid medium for 6 h (using the same protocol as for the in vitro pollen germination analyses). The germinated pollen was subsequently centrifuged and pollen pellets were stored using 100 µl RNAlater™ Stabilization Solution (Thermo Fisher Scientific, Waltham, MA, USA) until the RNA extraction.

For the non-sexual tissues, approximately 60 mg of leaf and root tissue was harvested and immediately frozen in liquid nitrogen until the day of RNA extraction. Just as for the other tissues, we collected material from one individual per mother plant and four mother plants per subspecies (2 subspecies × 1 individual × 4 mother plants = 8 samples per tissue). Total RNA was extracted using the protocol from MagMAX™ Plant RNA Isolation Kit (Thermo Fisher Scientific, Waltham, MA, USA) for all the tissues. The quality of the total RNA extracted from these tissues was quantified by Qubit (Thermo-Fisher Qubit 2.0 Fluorometer), and RNA concentration exceeded 72 ng/μl for all the samples. Library preparation and mRNA enrichment of the extracted RNA were prepared at the SEQme company in Dobříš, Czech Republic, using the QuantSeq 3ʹ mRNA-Seq Library Prep kit (Lexogen, Vienna, Austria). Finally, single-end libraries were sequenced at 100-bp read length using an Illumina NovaSeq6000 SP (**Supplementary Table S3**).

Endosperm RNAseq data used in this study were included to filter out seed-originated RNA from the pollinated pistils, as early seeds may have already been formed. Also, we included endosperm data to widen the range of tissue identities as comparison points to find tissue-specific genes. Due to the difficulty of isolating endosperm/early seed tissues, we obtained RNAseq data from a previous study conducted in *A. lyrata* (Klosinska et al. 2016) and downloaded from the Sequence Read Archive (GEO: GSE76076). The RNAseq data of five outcrosser endosperm samples were included in the data processing starting from the trimming step. While these data were only from outcrossers, we do not expect tissue-specificity expression patterns to significantly vary between the two subspecies. To make sure this is the case, we identified pollen-specific genes separately in selfers and outcrossers and verified that most of the genes show a similar pattern in both subspecies (**Supplementary Fig. S7**).

### Read trimming, mapping and counting

The quality of the raw RNA-seq reads was checked using FastQC ver. 0.11.9 (http://www.bioinformatics.babraham.ac.uk/projects/fastqc/). Before alignment, over-represented TruSeq adapters were trimmed using CUTADAPT ver. 3.5 (Martin 2011). Subsequently, we used trimmomatic ver. 0.39 (Bolger et al. 2014) to remove adapters and low-quality data with the following parameters: ILLUMINACLIP:TruSeq3-SE.fa:2:30:10, LEADING:3, TRAILING:3, SLIDINGWINDOW:4:15 and MINLEN:36. The trimmed reads were then mapped to the *A. lyrata* genome ver. 2.1 (Rawat et al. 2015) using the STAR alignment tool ver. 2.7.0a (Dobin et al. 2013) with option --outFilterMultimapNmax 1. This option retained only uniquely mapped reads in the output BAM file for subsequent analysis. Uniquely aligned reads were counted using the HTSeq count tool ver. 0.11.1 (Anders et al. 2015) with default parameters. *Arabidopsis lyrata* ver. 2.1 genome annotation (Rawat et al. 2015) was used for counting uniquely mapped reads.

### Differential gene expression and GO enrichment analysis

Before conducting the DEA, data normalization was performed based on sequencing depth and composition to assess sample quality according to their expression profiles. For this purpose, the vst normalization (variance stabilizing transformation) function from the DESeq2 package ver. 1.34.0 was used (Love et al. 2014). In order to see the profile of gene expression of each sample, normalized read counts among each replicate were visualized using principal component analysis (PCA) with the ‘plotPCA’ DESeq2 function, and a cluster dendrogram using the ‘hclust’ DESeq2 function in R (**Supplementary Fig. S1**).

DEA was performed using the DESeq2 tool ver. 1.34.0 in R (Love et al. 2014). To identify tissue-specific genes, we performed a DEA against other tissues. We considered genes as tissue-specific if they were upregulated (log_2_FoldChange ≥ 1 and adjusted *P* < 0.05) in all tissue pairwise comparisons. We chose a log_2_FoldChange threshold of 1 (i.e. a 2-fold change) to be sure of enough expression level differences between tissues for genes we call ‘tissue-specific’. For DEA between selfers and outcrossers, we used a different fold change threshold, |log_2_FoldChange| ≥ 0.5 (1.5-fold change) with adjusted *P* < 0.05 (**Supplementary Table S4**). We used this threshold to focus on small differences in gene expression between genetically closely related sister lineages. It is likely that small differences in gene expression can hold significant biological importance, especially when the impact of these differences is magnified through their influence on numerous genes (Huang et al. 2010). To compare the gene sets identified by DEA, Venn diagrams (Heberle et al. 2015) were generated for both tissue-specific and mating type–specific pairwise comparisons of the DEGs (**Supplementary Fig. S2**).

Biological roles of the obtained list of genes were identified with GO enrichment analysis (**Supplementary Fig. S3**). The GO enrichment analysis was performed using the PlantRegMap online tool (Tian et al. 2019). To improve the GO annotation of our genes, we decided to use *Arabidopsis thaliana* homologs of *A. lyrata* genes for enrichment analysis. We retrieved *A. thaliana* homologs from the *A. lyrata* ver. 2.1 annotation (Rawat et al. 2015). We used the list of these homologs as a background gene list to reduce the bias due to different genomes and gene content between the two species. By default, topGO and Fisher’s exact tests were applied to find the significantly overrepresented GO terms. Enriched GO terms corrected by parameters from the Benjamini and Hochberg method for multiple comparisons (FDR <0.05, *q* < 0.05) were considered as significant output. The *q*-value in the output file is defined as a natural FDR (positive FDR) analog to the *P*-value. The significance level of *q*-values (*q* < 0.05) was selected and transformed to negative fold changes (−log10) to present results in a bar chart. The bar charts were generated using the SRplot online platform (Tang et al. 2023).

## Supplementary Material

pcae090_Supp

## Data Availability

The RNA-Seq data used in this study have been submitted to the NCBI BioProject database (https://www.ncbi.nlm.nih.gov/geo/) under submission number PRJNA1067428.
