# Supplementary material for: Natural Variation in Sexual Traits and Gene Expression between Selfing and Outcrossing Arabidopsis lyrata Suggests Sexual Selection at Work
Source: Plant Cell Physiol. 2024 Aug 10;66(4):581–95. doi: 10.1093/pcp/pcae090 (PMC12085089; doi:10.1093/pcp/pcae090)
Supplement: pcae090_Supp [file pcae090_supp.zip › suppl_data/pcp-2024-e-00075-File008.docx]

**
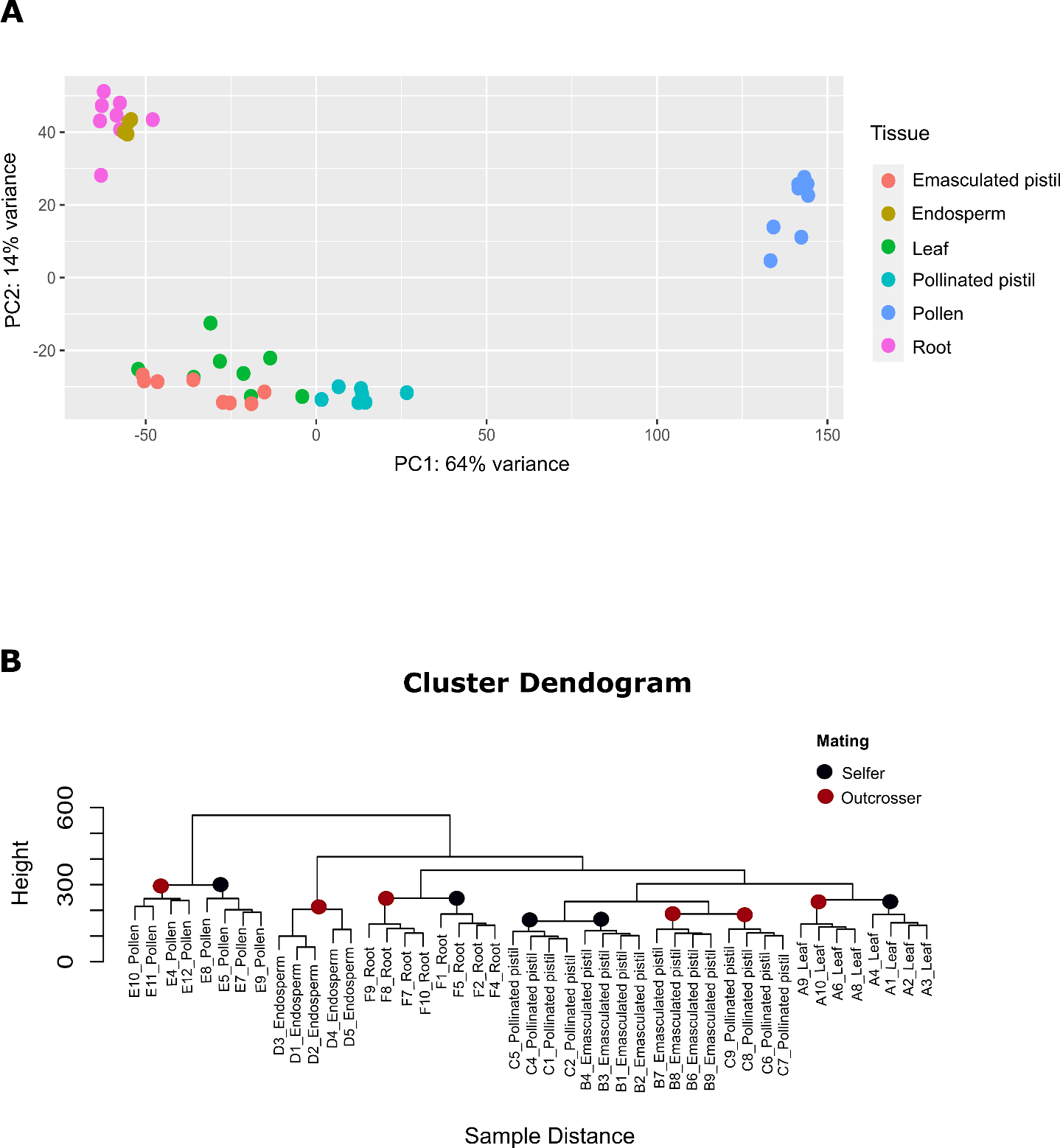
Figure S1: General expression patterns of samples depending on tissue type and population.** The variability of gene expression patterns among the samples was visualized with principal component analysis (PCA) (A), and dendrogram clustering (B). PC: principal component. In B), names such as "A1" are sample codes, described in detail in Table S3.

**
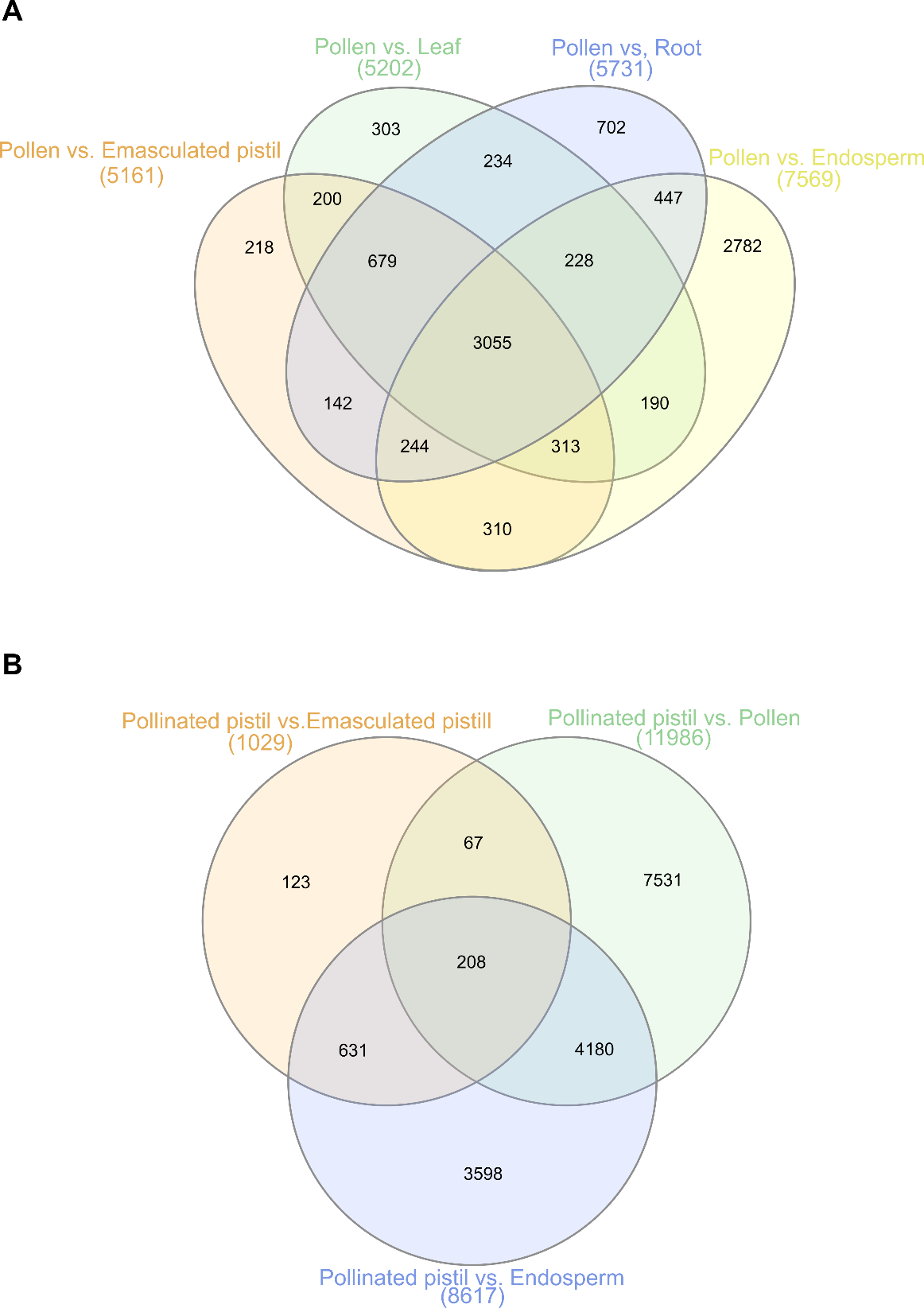
**

**Figure S2: Venn diagram showing the number of tissue-specific genes in pollen (A), and pollinated pistil (B).**

**
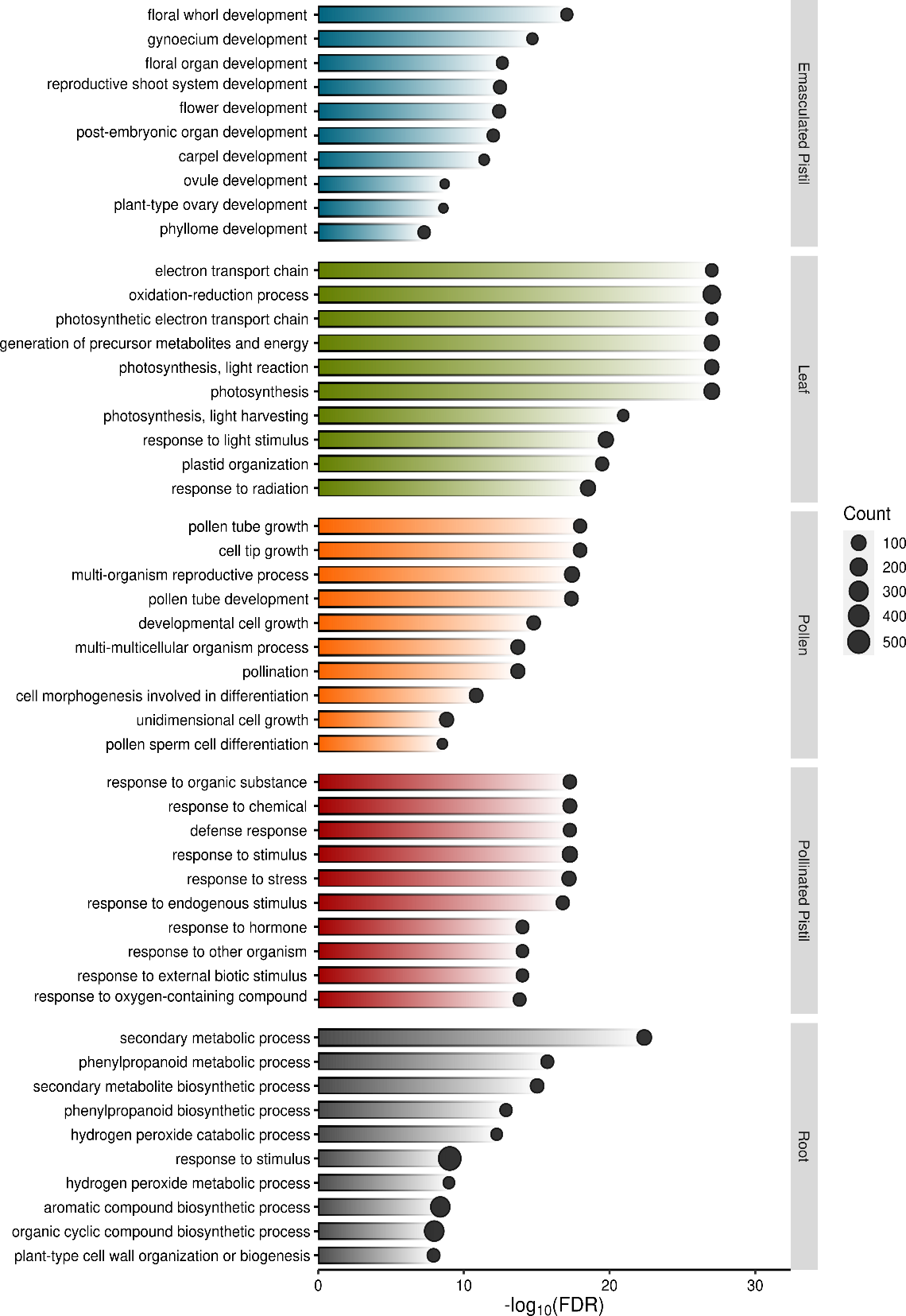
**

**Figure S3: Biological validation of tissue-specific genes among the samples sequenced in this study**. Tissue-specific DEGs were used as an input gene set for the gene enrichment analysis. All GO terms are the categories of biological processes. Count indicates the number of genes that were significantly enriched in respective terms.

**
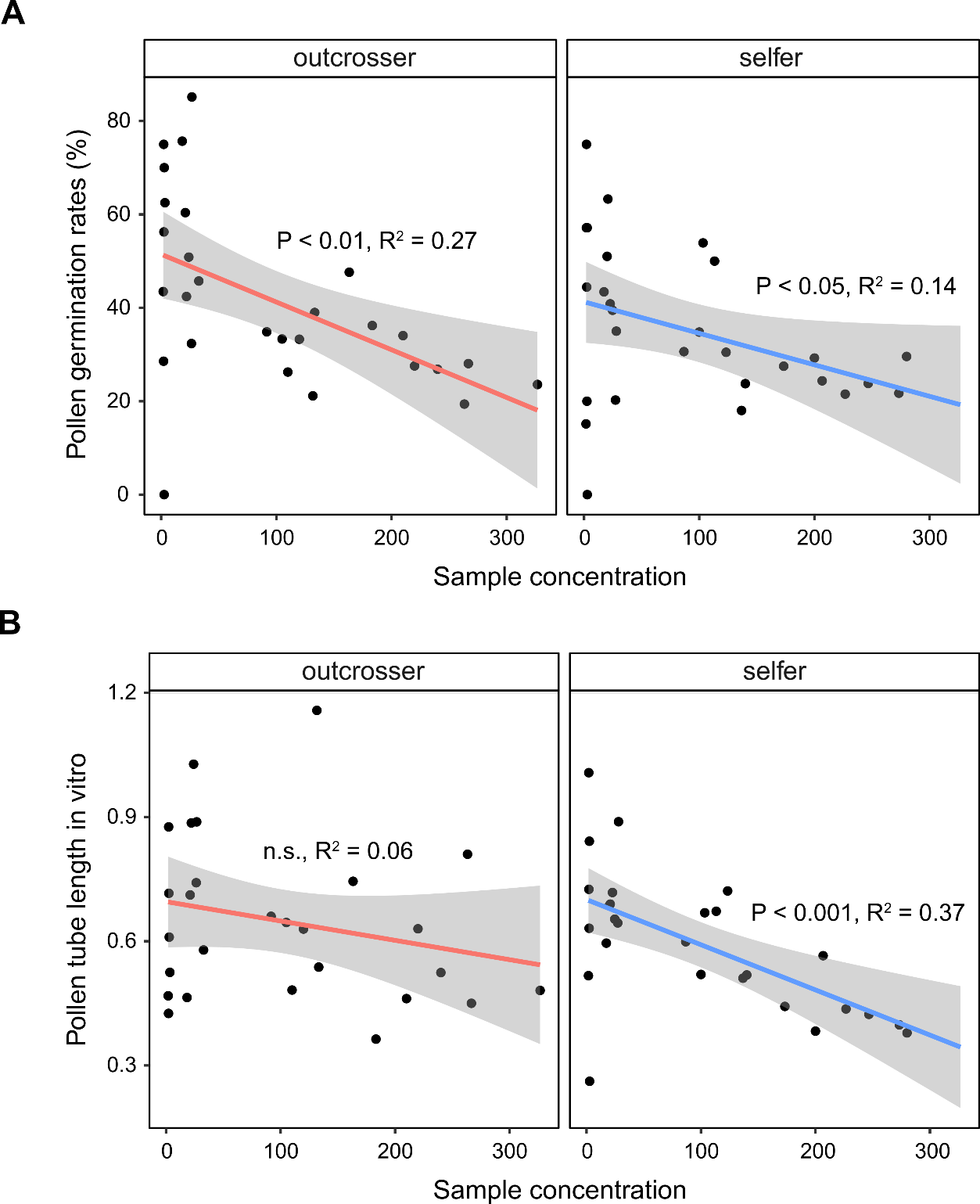
**

**Figure S4: Different performance of pollen originating from outcrosser and selfer individuals of *Arabidopsis lyrata* in a pilot experiment.** Pollen germination rates (A) and pollen tube growth (B) were assessed under experimentally manipulated gradients of pollen concentration after 16 hours.


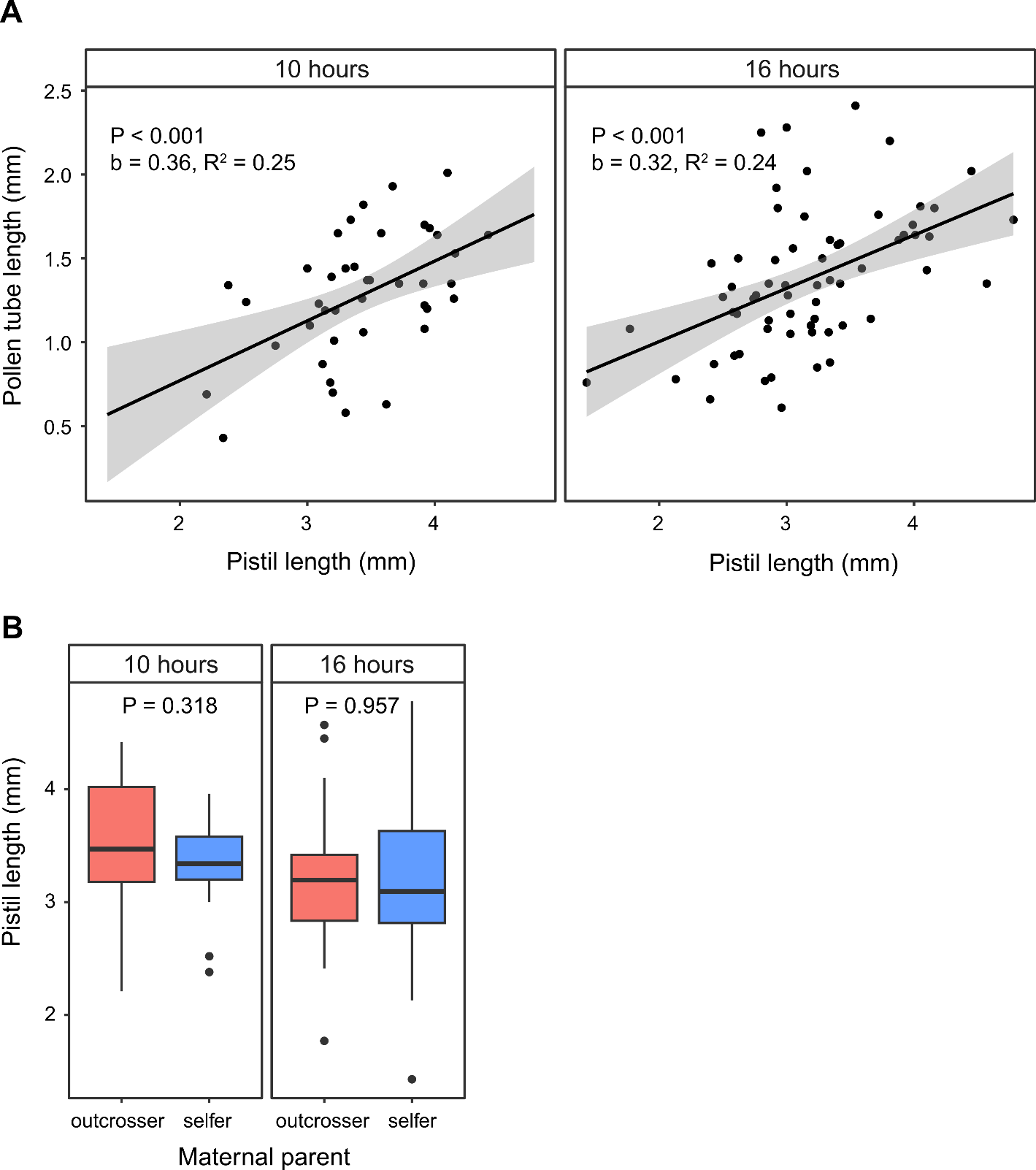


**Figure S5: Dependence of pollen tube length on pistil length (A) and no difference in mean pistil length between outcrossers and selfers of *Arabidopsis lyrata* (B)**. Comparisons were made independently for two experiments that differed in the time allowed for pollen germination and growth (10h vs. 16h).


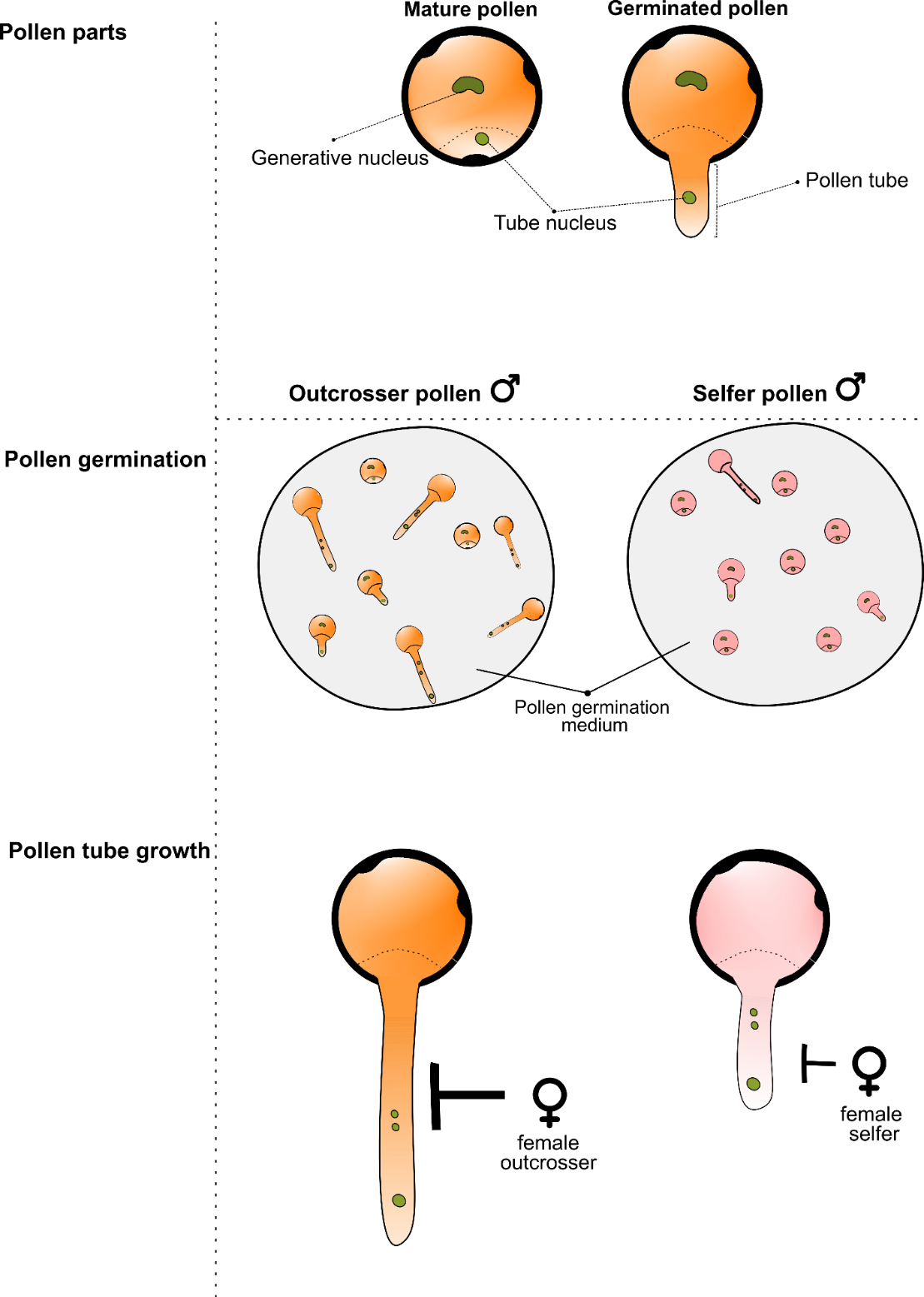


**Figure S6: Summary figure depicting the pollen germination and pollen tube growth differences between selfing and outcrossing *A. lyrata*.** Black block bars in pollen tube growth part represent the strength of female influence on pollen tube growth from outcrosser and selfer *A.lyrata*. The size of the block bar correlated with the strength of female influence, the bigger the stronger.


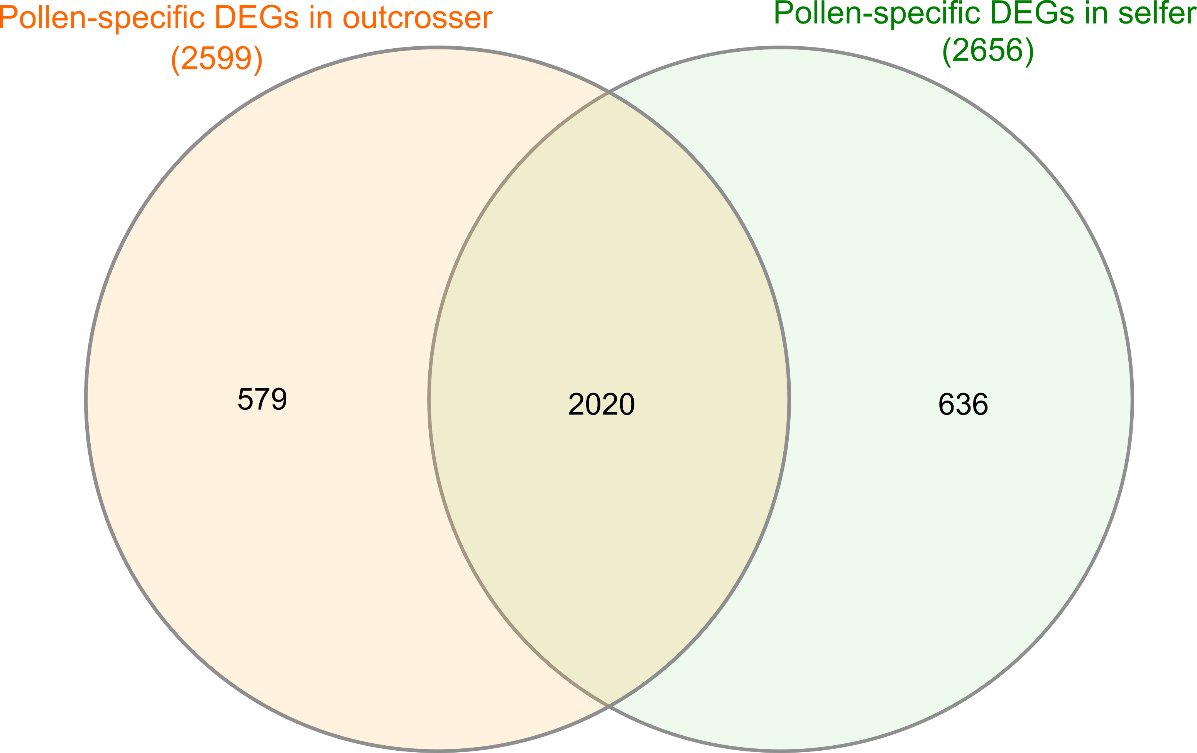


**Figure S7: Venn diagram showing the overlap between pollen-specific genes separately identified in outcrossers and selfers of *Arabidopsis lyrata*.**

**Table S1**: In vitro pollen germination and pollen tube growth dataset

**Table S2.** In vivo pollen tube growth dataset

**Table S3.** Population index and RNAseq library information

**Table S4.** List of tissue and mating-specific genes identified in this study

**Table S5:** Summary of a linear mixed-effects model run on the combined dataset of two crossing experiments differing in the time allowed for pollination (10h vs. 16h), testing the effects of mating system (outcrosser vs. selfer) of the maternal parent, mating system of the paternal parent, and their interaction on pollen tube growth in vivo. Seed families of the maternal and paternal parents were used as random effect factors.

| **Response: Pollen tube growth in vivo** |  |  |  |
| --- | --- | --- | --- |
|  | **d.f.** | **F** | **P** |
| Pollination time | 1, 16.9 | 6.4 | **0.022** |
| Maternal mating system (MMS) | 1, 3.1 | 3.1 | 0.175 |
| Paternal mating system (PMS) | 1, 1.3 | 0.7 | 0.526 |
| MMS × PMS | 1, 89.6 | 5.0 | **0.027** |

**Table S6.** Detailed output of gene ontology results from pollen-specific and pollinated-specific genes with divergent expression between selfing and outcrossing *A.lyrata,*
